# Preparation of colourless phosphate glass by stabilising higher Fe[II] in microwave heating

**DOI:** 10.1038/s41598-018-24287-1

**Published:** 2018-04-18

**Authors:** Ashis K. Mandal, B. Mandal, Kavya Illath, T. G. Ajithkumar, A. Halder, P. K. Sinha, Ranjan Sen

**Affiliations:** 1 CSIR-Central Glass and Ceramic Research Institute, 196, Raja S. C. Mullick Road, Kolkata, - 700032 India; 2 Central NMR Facility and Physical and Materials Chemistry Division, CSIR-National Chemical Laboratory, Dr.Homi Bhabha Road, Pune, - 411008 India

## Abstract

Iron impurity in raw material remains a major challenge in producing colourless glass. In this investigation, we report microwave (MW) heating capable of altering Fe-redox ratio (Fe^2+^/∑Fe) enabling preparation of colourless phosphate glass. The effect of Sn concentration in retention of Fe[II] in glass melted in MW was compared with conventional glasses. Colourimetric study developing Fe^2+^-ferrozine colour complex reveals Fe-redox ratio ≥0.49 required to obtain colourless phosphate glass. In microwave heating, addition of 1 wt.% Sn metal powder can impart the desired effect whereas addition of 1.9 wt.% Sn metal powder is required in conventional heating. The correlation equation of Fe-redox ratio with concentration of Sn metal is found to be different in microwave and conventional heating. Thus, exploiting this different redox changes in MW heating optical properties can be tailored. Preservation of higher Fe[II] in MW melted glass is also confirmed by XPS and TGA. ^31^P MAS NMR spectra suggest that transition from cross linked ultra phosphate to linear polymer metaphosphate network in incorporation of Sn is found different in glass prepared adopting microwave irradiation. ^27^A1 MAS NMR spectra suggest higher relative content of Al^6+^ in glass obtained from MW heating. Energy consumption analysis revels 3.4 kWh in MW heating while 14 kWh in conventional glass melting using resistance heating. Further, glass melting in MW can be completed within 2 h unlike ~5 h needed in conventional. MW heating plays a significant role in improving properties to make colourless phosphate glass in addition to significant energy and time saving.

## Introduction

Iron plays an important role in glass making as a colourant either by deliberate addition or as impurity. The choice of raw material is largely governed by iron content. Often, very minor amount of iron produce colour in glass. The selection of refractory material for construction of furnace and crucible material depend on its iron content and the risk of contaminating the glass. Thus, despite many precautions iron enter the glass melt either from furnace atmosphere or from raw material. Property of glass not only depends on the concentration of iron, but its oxidation state also. General consensus about the presence of iron in glass as Fe^2+^ and Fe^3+^, both of which can exist in tetrahedral and octahedral sites^1–5^. Each redox and coordination can produce distinct optical absorption bands. Majority of Fe^3+^ ions are supposed to occupy tetrahedral network-forming sites^6–9^, but Fe^2+^ ions are believed to occupy octahedral network-modifying sites^10,11^.

The ferrous ion in aqueous solution is colourless with characteristic near infra-red (IR) absorption band^12^. The light absorption of the ferric ion seems to be more complicated than that of ferrous ion. A ferric ion has a strong ultra-violet absorption which reaches into the visible ranges of the spectrum, resulting yellow or brown colour. Although iron present in combination with Fe^2+^ and Fe^3+^ in glass melt, trivalent state is predominate under oxidizing atmosphere melting, resulting yellow or brown colour in glass. Colour of glass depends on many factors including iron-redox ratio (Fe^2+^/∑Fe). The colour of glass becomes bluish green with increasing Fe-redox ratio and turns yellow or brown with lower Fe-redox ratio. In conventional glass melting under air atmosphere, Fe- redox ratio remains low signifying more retention of trivalent oxidation state and thus makes the glass yellow or brown in colour.

Microwave (MW) heating offers several advantages in material processing due to its fundamental difference in heating mechanism^13,14^. Several materials are prepared with improved properties using MW energy^15,16^. The main advantages of this melting method remain energy efficiency and shorter melting time^17^. However, improved properties and low contamination from the crucible wall have also been observed in glasses prepared adopting microwave radiation^18–20^. Retention of higher ferrous oxidation state in phosphate glass has also been reported in MW heating^21^.

The objective of this study is to investigate preparation of colourless phosphate glass containing Fe in MW heating. Iron containing P_2_O_5_- Al_2_O_3_-Na_2_O glass system was melted with varying content of Sn metal in both the methods. Sn concentration was optimized in MW heating to produce colourless glass for the application of heat absorbing screen. Determination of threshold Fe-redox ratio required to make the glass colourless is another aim of this investigation.

## Results

**Figure 1:**
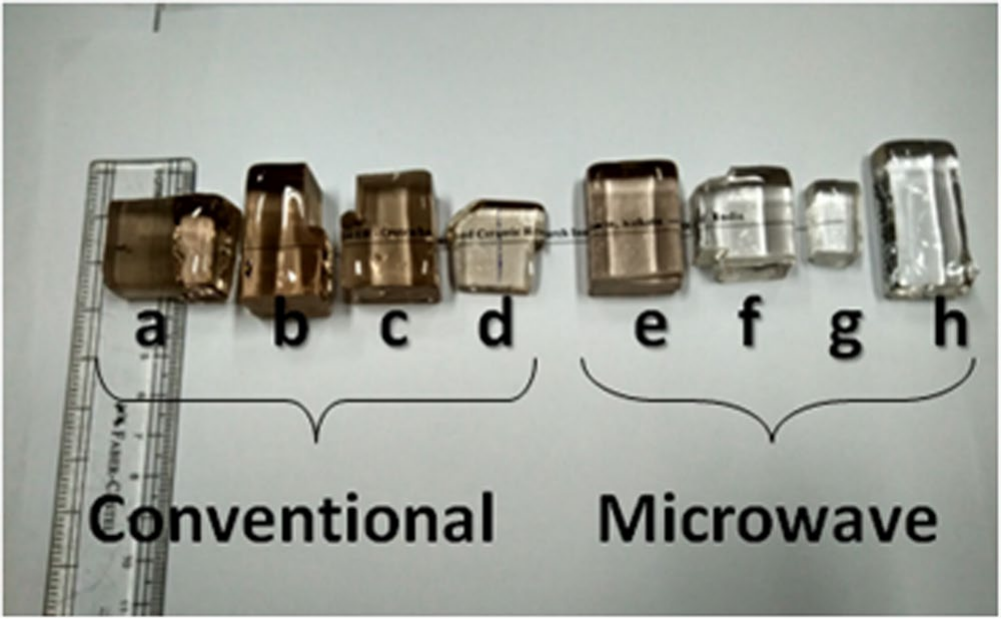
Photograph of Fe-doped phosphate glasses melted in both conventional and MW heating with increasing concentration of Sn (0 to 1.5 wt.%) [a: GC 0Sn, b: GC 0.5Sn, c: GC 1Sn, d: GC 1.5Sn, and e: GM 0Sn, f: GM 0.5Sn, g: GC 1Sn, h: GM 1.5Sn].

Figure 1 displays photograph of all the annealed glasses obtained from both the heating methods. Two sets of four glasses with varying Sn (reducing agent) concentration 0, 0.5, 1 and 1.5 (wt.%) are shown from left to right [a: GC 0Sn, b:GC 0.5Sn, c: GC 1Sn, d: GC 1.5Sn, and e: GM 0Sn, f: GM 0.5Sn, g: GM 1Sn, h: GM 1.5Sn]. [Composition and melting method are mentioned in Material and Method section]

**Figure 2:**
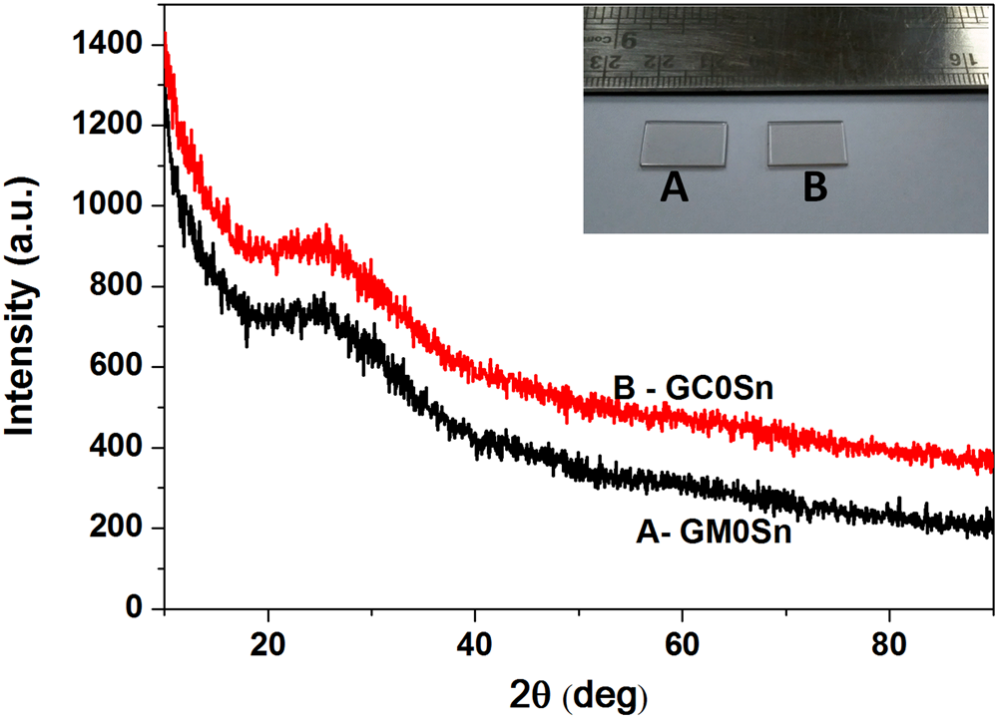
XRD profile for (A) GM 0Sn and (B) GC 0Sn samples.

Figure 2 depicts X-ray diffractograms (XRD) for GC 0Sn and GM 0Sn samples. A broad hump centred around 2θ = 25° is seen in both the XRD profile of GM 0Sn and GC 0Sn. The polished glass samples of 0.5 mm thickness GM 0Sn (A) and GC 0Sn (B) are shown in the inset of Fig. 2.

**Figure 3:**
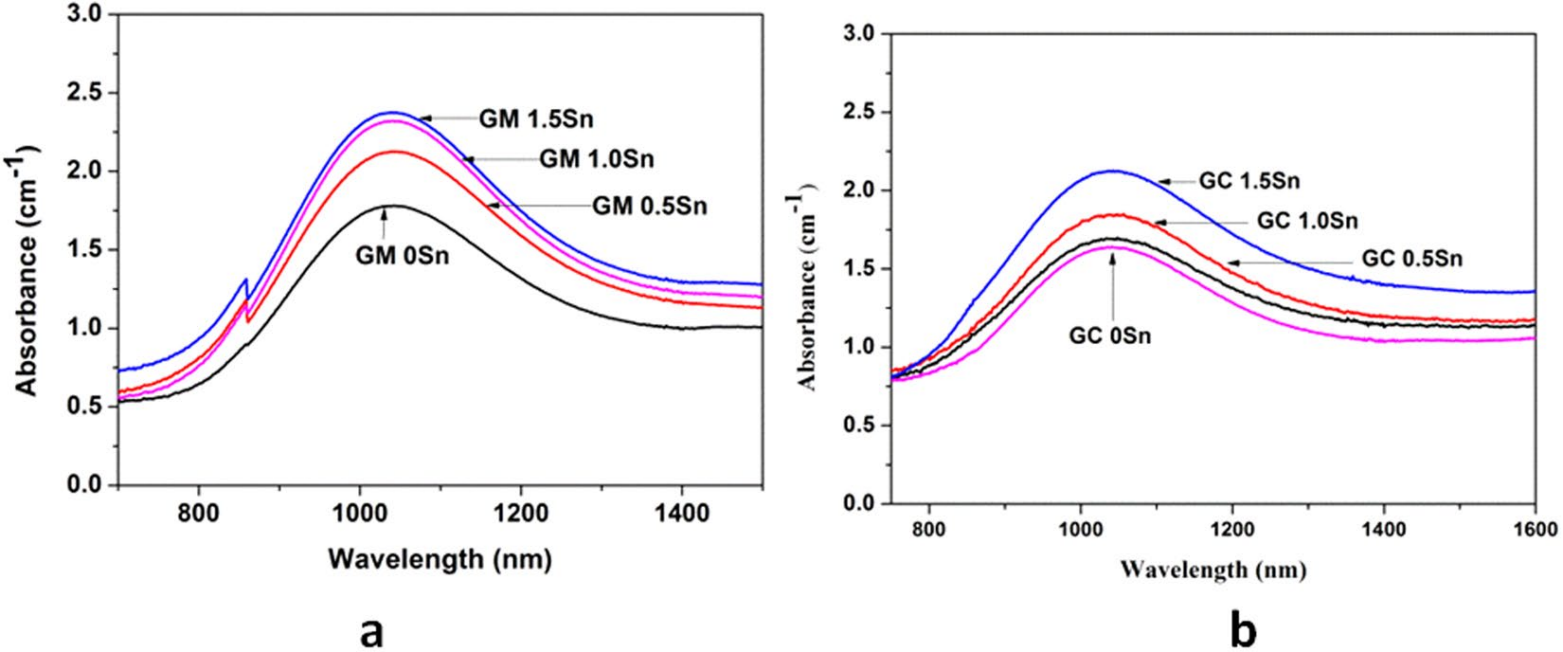
Vis-NIR absorbance spectra of all the studied glasses obtained from (**a**) MW heating; (**b**) conventional heating.

Vis-NIR absorbance spectra recorded for the glasses (GM 0Sn, GM 0.5Sn, GM 1.0Sn and GM 1.5Sn) obtained in MW heating have been illustrated in Fig. 3a showing broad absorbance band centred at ~1050 nm, which is ascribed to the ferrous ions in glass. Similar broad peak at ~1050 nm in Vis-NIR absorbance spectra of conventional glasses (GC 0Sn, GC 0.5Sn, GC 1Sn, GC 1.5Sn) has been identified in Fig. 3b.

**Figure 4:**
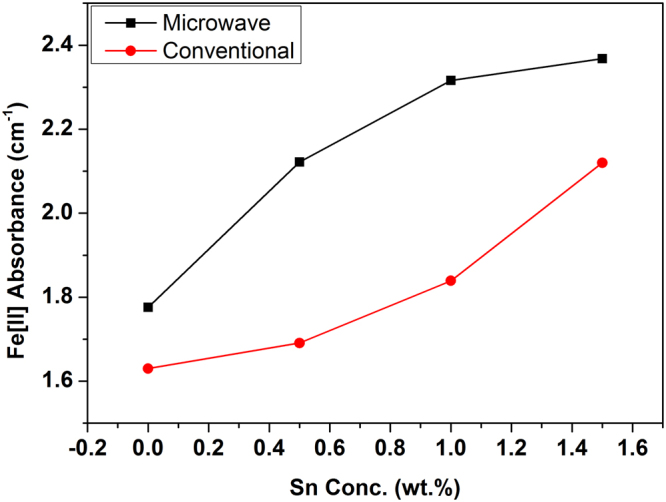
Comparison of ferrous absorbance (at ~1050 nm) in glass with varying concentration Sn (0 to 1.5 wt.%) in both method. (Lines are drawn to guide the eye).

The absorbance of Fe[II] (in cm^−1^) with concentration of Sn in the glasses prepared in both the heating routes is plotted and exhibited in Fig. 4. The absorbance intensity of ferrous ions in base glass (i.e without Sn metal) melted in MW heating is found higher than that of conventional glass. It has been observed that addition of 0.5% Sn metal improves the Fe[II] absorbance intensity distinctly in MW altering the visible colour; whereas addition of 0.5 wt.% Sn makes insignificant changes in conventional glass showing no visible colour change. Further, addition of 1 wt.% Sn metal in glass enhances the Fe[II] absorbance to 2.31 cm^−1^ and the glass is visibly colourless in MW.

**Figure 5:**
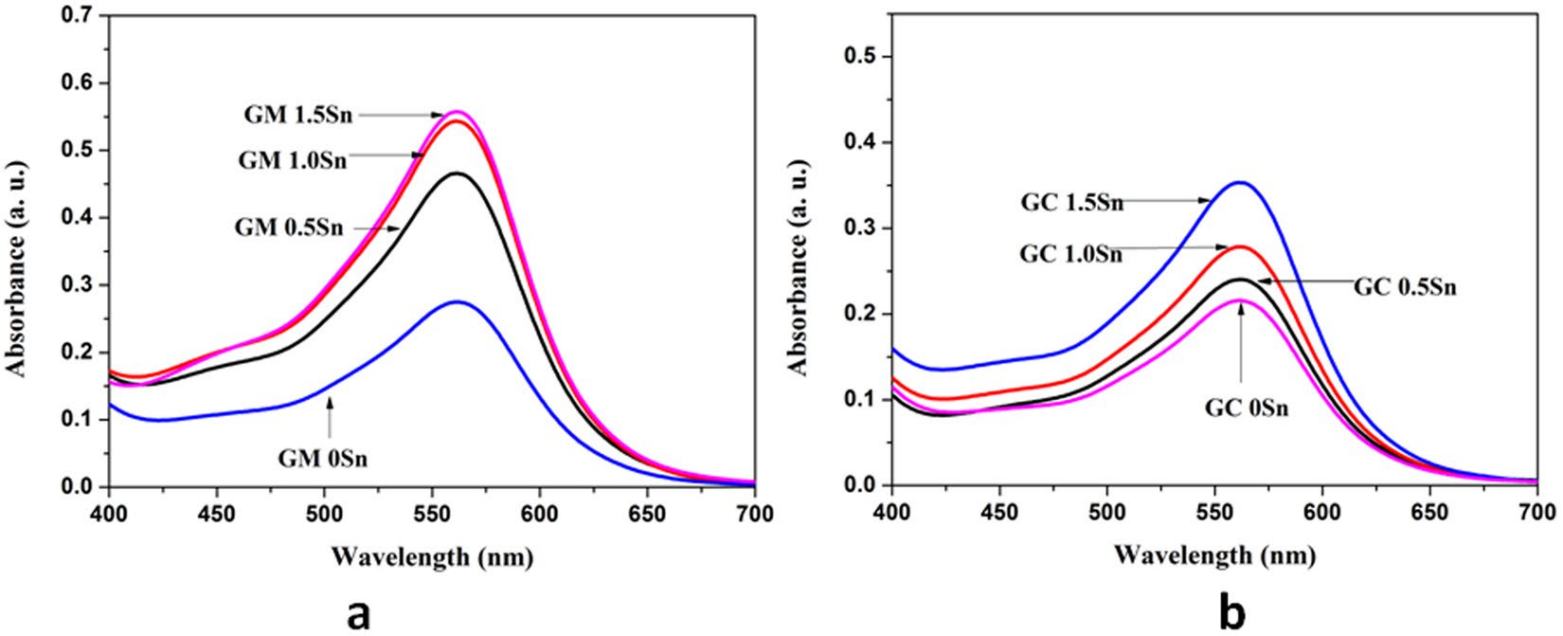
Visible absorbance spectra depicting broad absorbance band centered at 562 nm for Fe^2+^-Ferrozine complex for the glass containing varying concentration Sn (0 to 1.5 wt.%); (**a**) MW heating; (**b**) conventional heating.

Figure 5a depicts visible absorbance spectra (within wavelength 450–650 nm) of Fe^2+^-Ferrozine complex in the solution developed from the glasses [GM 0Sn, GM 0.5Sn, GM 1Sn and GM 1.5 Sn] prepared in MW heating. Similarly, Fig. 5b portrays visible absorbance spectra of Fe^2+^-Ferrozine complex in the solution developed from the conventional glasses [GC 0Sn, GC 0.5Sn, GC 1Sn, GC 1.5Sn]. Although absorbance intensity at 562 nm increases with increasing concentration of Sn, increase of this intensity for GC 0.5Sn is recorded much less than that of GM 0.5Sn.

**Figure 6:**
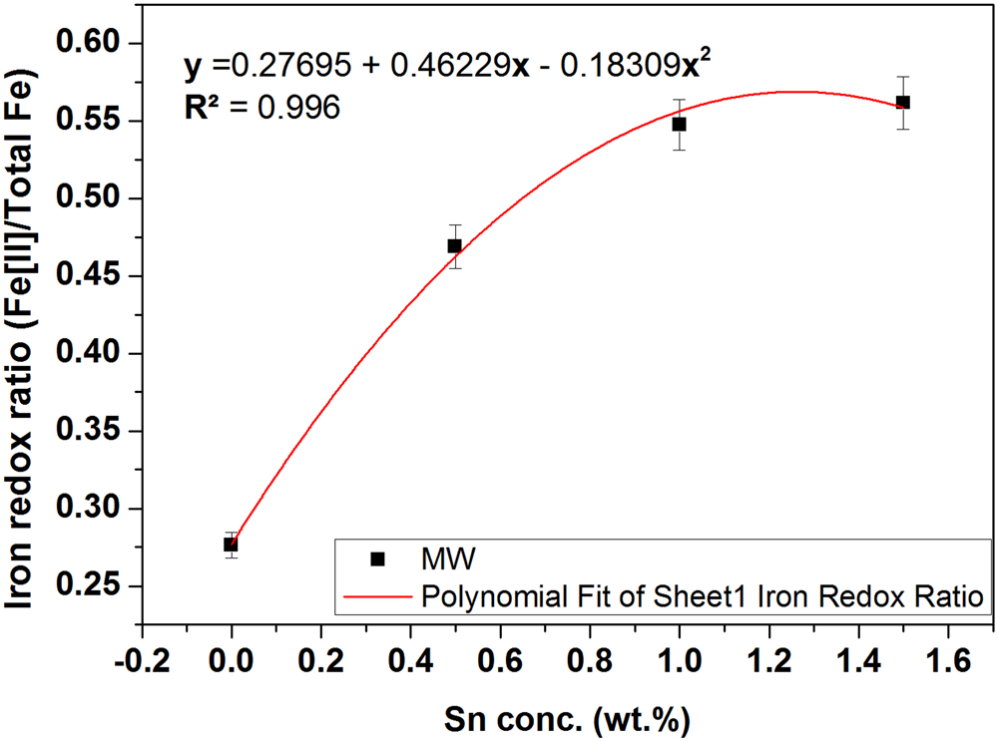
Fe-redox ratio [Fe^2+^/∑ Fe] in the glasses with varying concentration of Sn in microwave heating.

Fe^2+^-Ferrozine complex in the solution to estimate total Fe was evaluated using GC 0Sn and GM 0Sn samples for both the methods. Total iron (Fe) was estimated using method as described elsewhere^22^. Three measurements were carried out and average value at 562 nm was recorded as relative total iron concentration in the glasses. Iron redox ratio [Fe(II)/∑Fe] is evaluated and plotted in Fig. 6 with varying Sn concentration in MW prepared glasses. In MW heating, the redox ratio is estimated 0.28, 0.45, 0.49, 0.53 for the glasses containing 0, 0.5, 1 and 1.5 wt.% of Sn, respectively.

**Figure 7:**
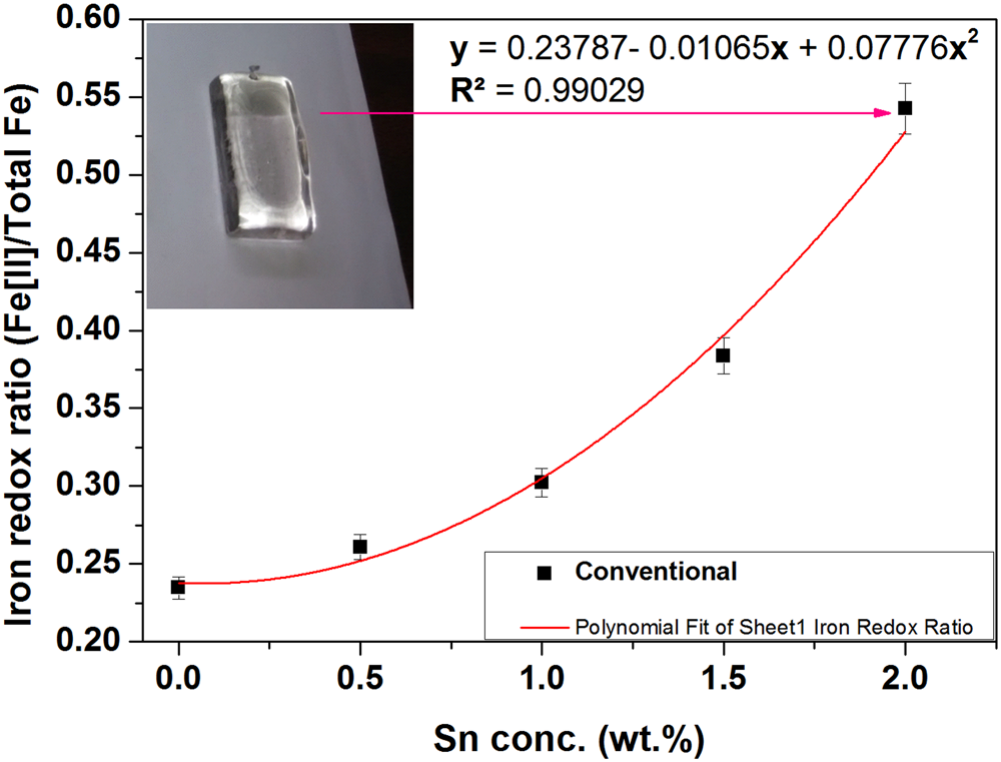
Fe-redox ratio [Fe^2+^/∑ Fe] in glasses with varying concentration of Sn in conventional heating.

Similarly, the Fe-redox ratio in conventional glasses is evaluated 0.23, 0.25, 0.28 and 0.37 for 0, 0.5, 1, and 1.5 wt.% addition of Sn, respectively. Now, in order to investigate the requirement of Sn to make the the glass colourless 2 wt.% of Sn metal was added and a glass was prepared in conventional heating. The Fe-redox ratio is estimated 0.54 in glass melted with addition of 2 wt.% Sn. Now, Fe-redox ratio is plotted with Sn concentration and displayed in Fig. 7.

**Figure 8:**
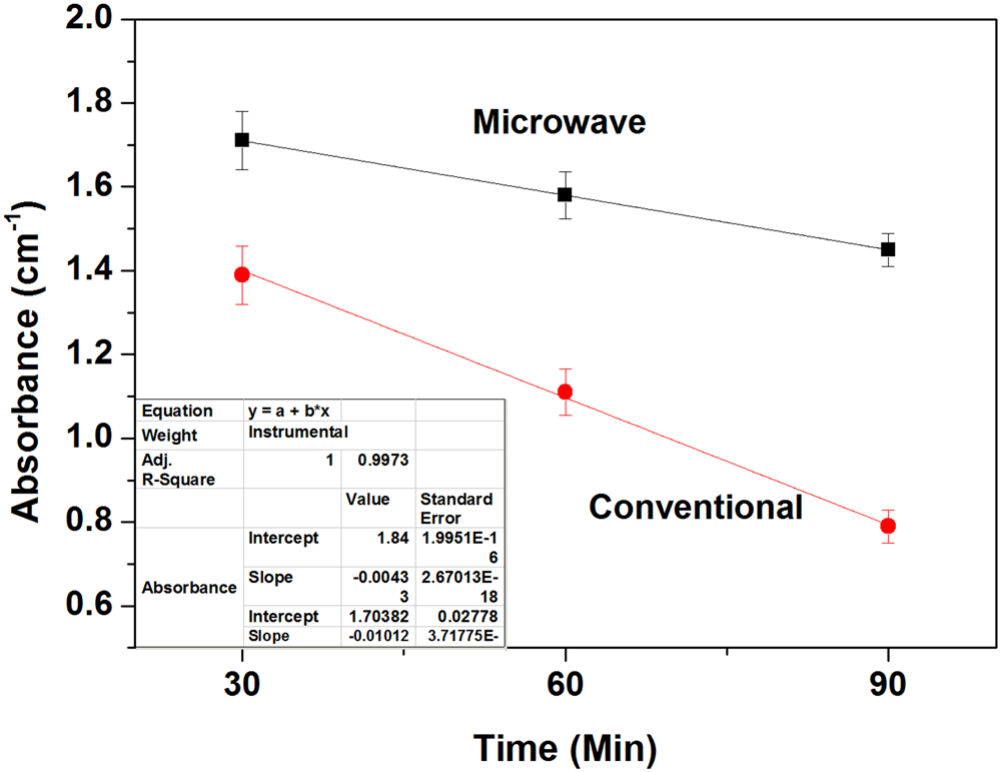
Fe[II] absorbance intensity of glass containing 0 Sn with varying melting time in both the heating method.

The base glass (without addition of reducing agent i.e. 0 Sn) was melted at identical temperature in microwave and in resistance heating furnace varying melting time at 30, 60 and 90 minutes maintaining similar condition to understand kinetics of Fe[III] formation. UV-Vis-NIR spectra were recorded for all the glasses of thickness 0.5 mm [not shown here] and absorbance (in cm^−1^) at 1050 nm is plotted with melting time in Fig. 8.

**Figure 9:**
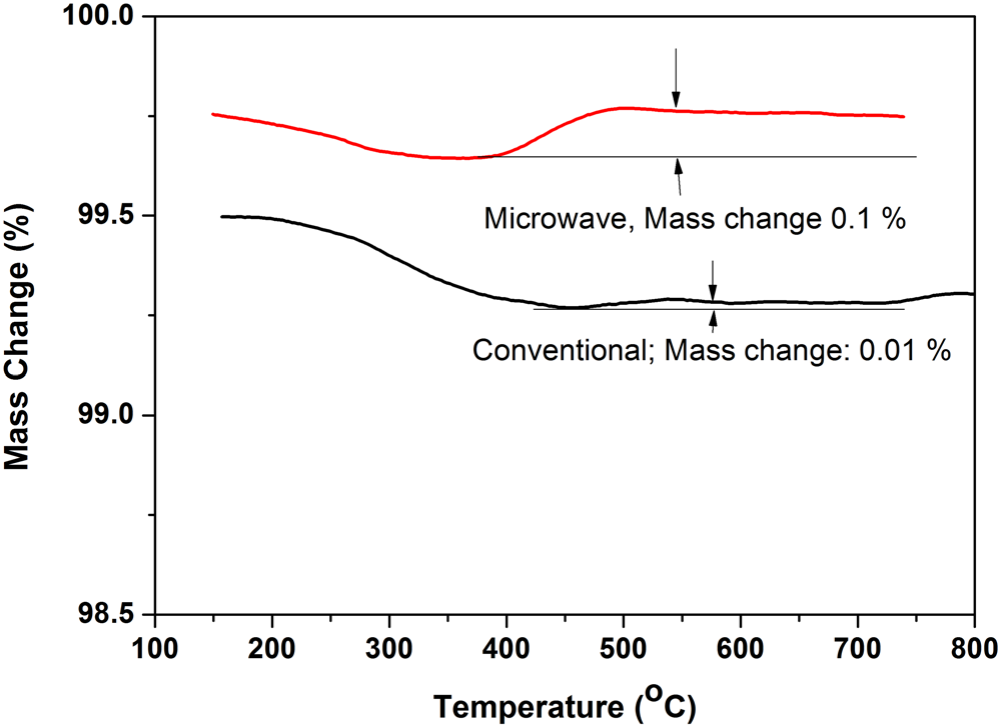
More weight gain in TGA profile signify higher retention of Fe[II] in microwave prepared glass.

Mass change in thermogravimetric analysis (TGA) of the glass melted with 5 wt.% Fe metal in both heating methods up to 800 °C is demonstrated in Fig. 9.

**Figure 10:**
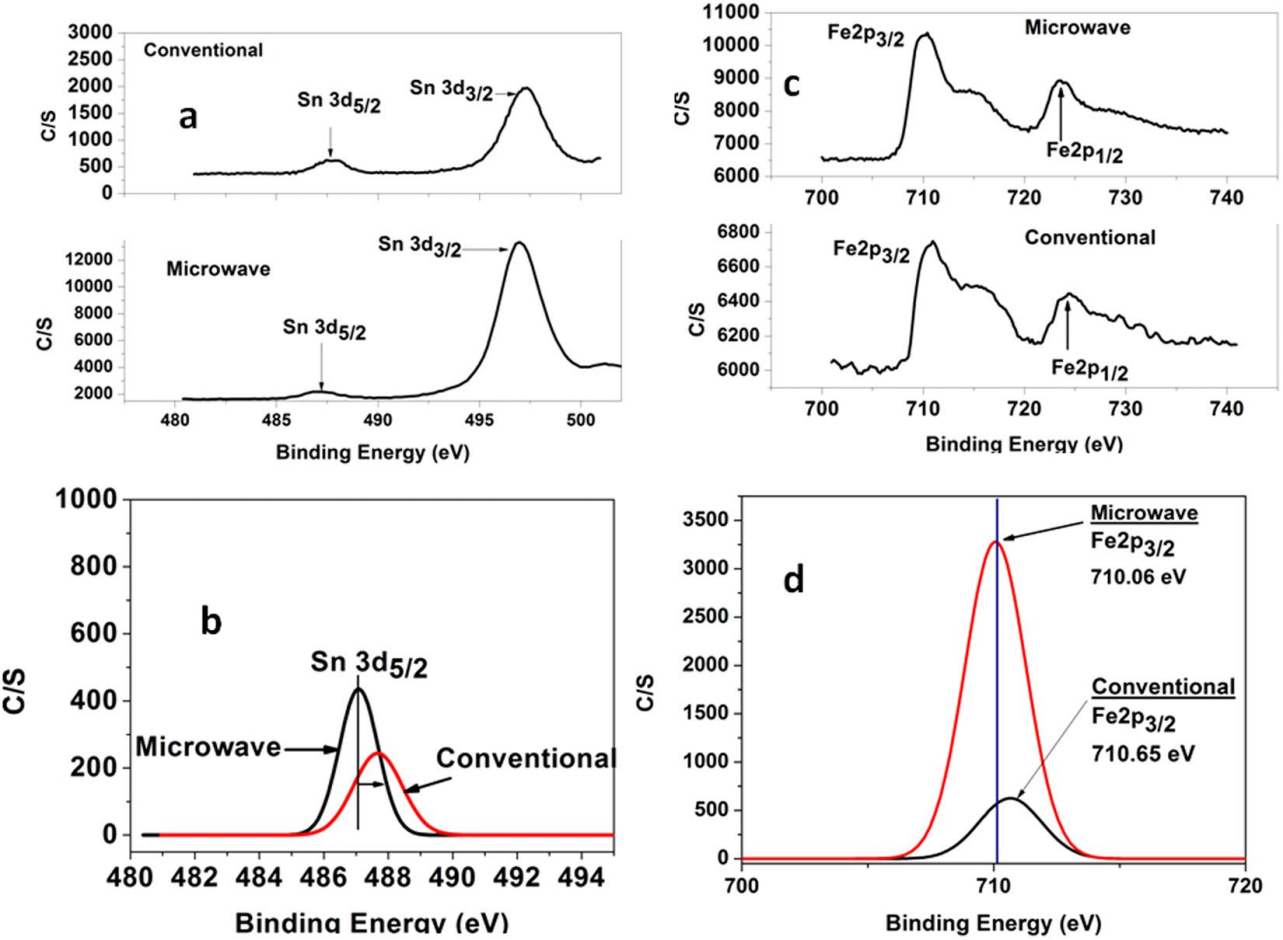
(**a**) Core level XPS spectra of Sn3d within 480–500 eV of GMF5 and GCF5, (**b**) Deconvoluted peaks of Sn3d_5/2_ at ~486 eV of GMF5 and GCF5. (**c**) Core level XPS spectra of Fe2p within 700–740 eV of GMF5 and GCF5, (**d**) Deconvoluted peaks of Fe 2p_3/2_ at ~710 eV of GMF5 and GCF5.

Figure 10 depicts core level scan X-Ray Photoelectron spectra (XPS) for GMF5 and GCF5. Figure 10a depicts core level Sn3d spectra of both the glasses. Deconvoluated spectra for Sn 3d_5/2_ at 487 eV is presented in Fig. 10b. Figure 10c depicts core level Fe2p spectra of both the glasses. Deconvoluted spectrum of Fe 2p_3/2_ at 710 eV for GMF5 and GCF5 is shown in figure Fig. 10d.

### Temperature–time -Power

**Figure 11:**
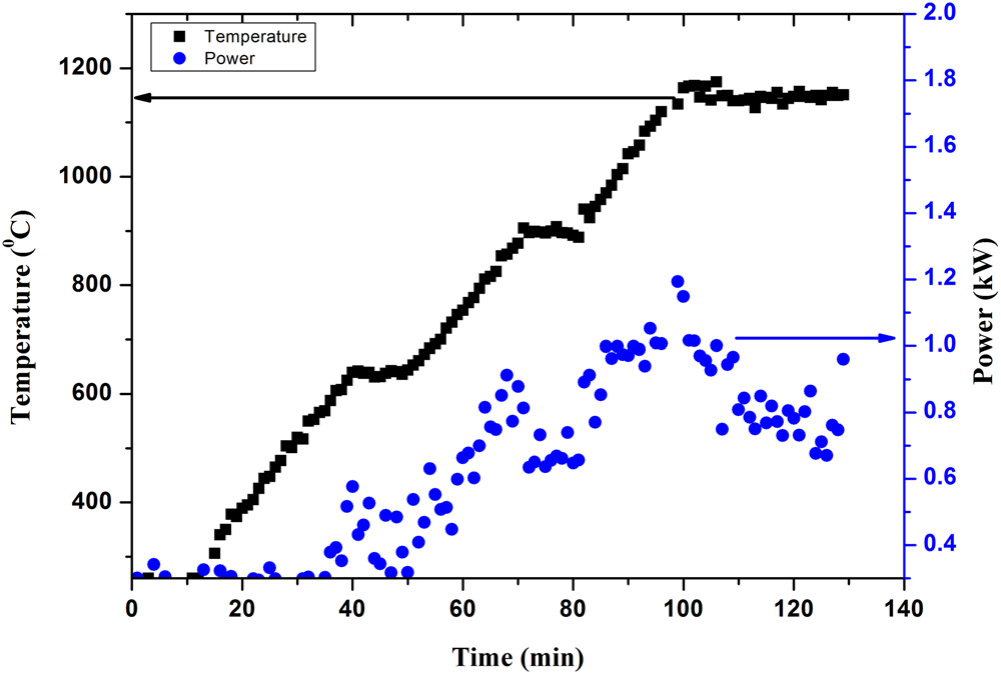
Temperature, MW Power and time (T-P-t) profile of glass melting in MW furnace.

Temperature, MW output power (MW power) and time (T-P-t) profile is shown in Fig. 11. The maximum MW power was recorded ~1.1 kW, as seen in Fig. 11. The actual input electrical power was within 1.7 kW (considering 70% magnetron efficiency). Total electrical power consumption was 3.4 kWh including electrical power load for utilities like chiller for cooling, diaphragm pump and control system. Moreover, total time, as seen in Fig. 11, needed in MW melting for this studied glass was 130 min.

**Figure 12:**
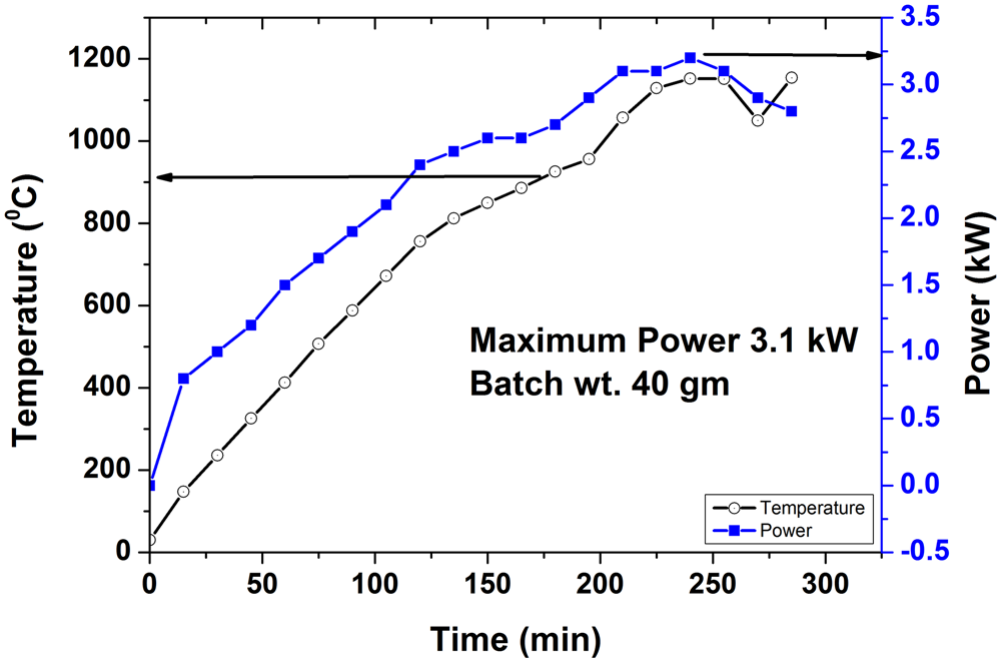
Temperature, Power and time (T-P-t) profile of glass melting in conventional resistance heating furnace.

The temperature, power and time profile is depicted in Fig. 12 for conventional melting. Maximum instantaneous electrical power demand was recorded more than 3 kW with total power consumption 14 kWh. It can be seen from Fig. 12 that total melting time is ~300 min (5 h) in contrast to 130 min (~2 h) in MW heating.

### ^31^P and ^27^Al NMR analysis

**Figure 13:**
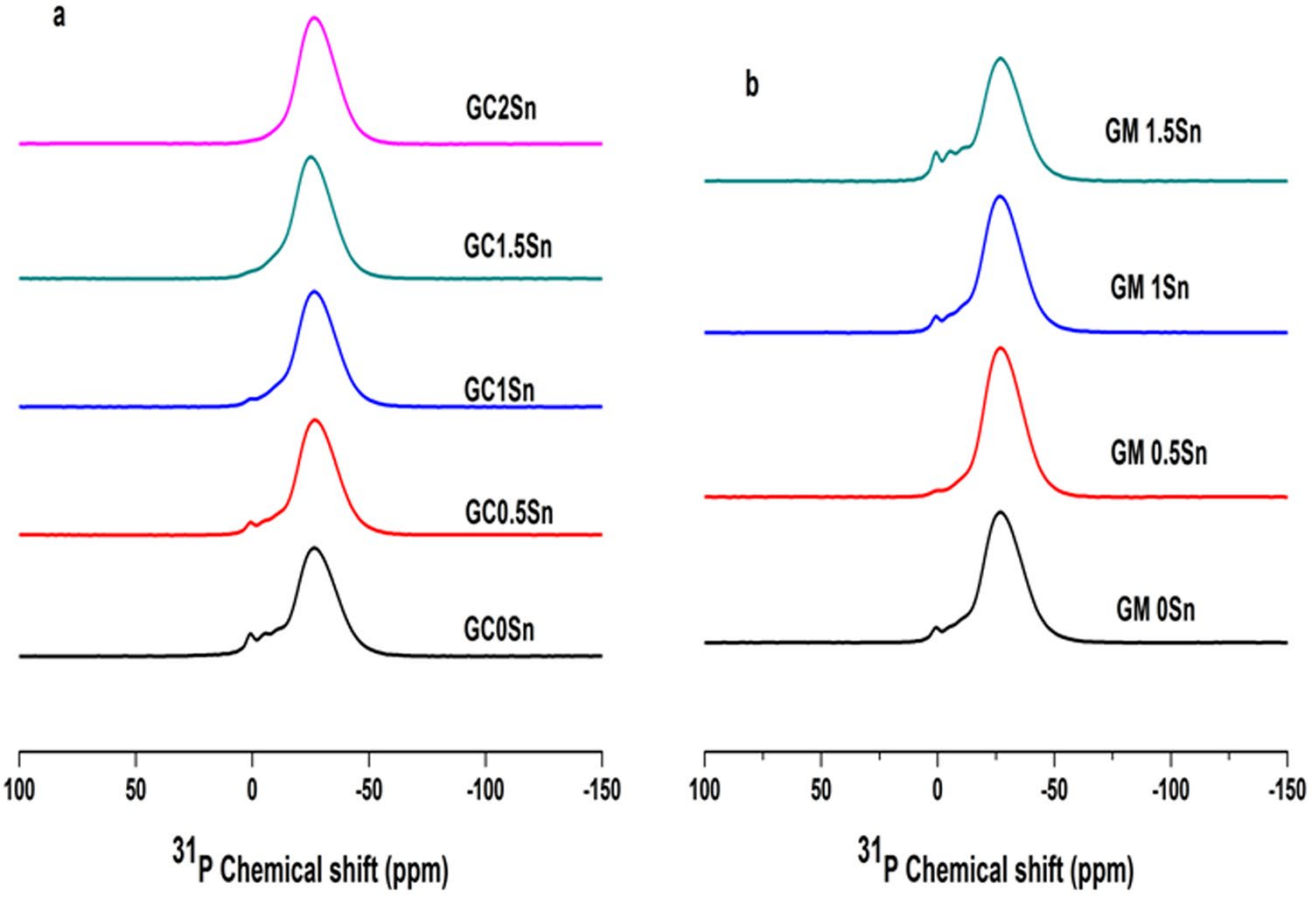
^31^P MAS NMR spectra for the glasses having different concentration of Sn.; (**a**) conventional heating; (**b**) MW heating.

The nature of the P-O network in the glasses could be obtained from a detailed analysis of the ^31^P MAS NMR spectra and is shown in Fig. 13. All the spectra have been deconvoluted into five components. The parameters obtained from the deconvolution are shown in the Table 1 and the peaks can be attributed to the Q^0^ (~1 ppm), Q^1^ (~−5 to −10 ppm), Q^2^ (−23 ppm) and Q^3^ (~−30 ppm) sites respectively^23^.

**Table 1:** Relative content of Q^n^ groups and their chemical shift obtained from ^31^P MAS NMR spectra after deconvolution.

Glass	Chemical shift (ppm)	% of occupancy	Glass	Chemical shift (ppm)	% of occupancy
GC0Sn	0.73	5.10	GM0Sn	0.95	1.97
	−4.90	2.05		−4.85	1.93
	−10.05	4.73		−10.43	2.77
	−23.68	30.32		−24.38	41.64
	−30.84	57.80		−32.15	51.68
GC0.5Sn	0.99	1.91	GM0.5Sn	1.07	0.33
	−4.91	2.01		−5.40	1.13
	−10.56	2.71		−10.61	1.56
	−24.02	36.93		−24.20	41.94
	−31.54	56.45		−32.07	55.04
GC1Sn	1.40	0.7	GM1Sn	0.91	1.98
	−6.07	3.02		−4.47	1.71
	−11.09	2.05		−10.20	3.52
	−23.88	41.71		−24.24	43.81
	−31.65	52.52		−31.95	48.48
GC1.5Sn	1.94	0.42	GM1.5Sn	0.74	4.60
	−5.21	2.97		−4.73	2.97
	−10.55	3.17		−10.04	5.25
	−22.76	44.38		−24.51	44.07
	−30.68	49.07		−32.34	43.11
GC2Sn	−0.63				
	−7.98				
	−11.72				
	−23.88				
	−31.94				

**Figure 14:**
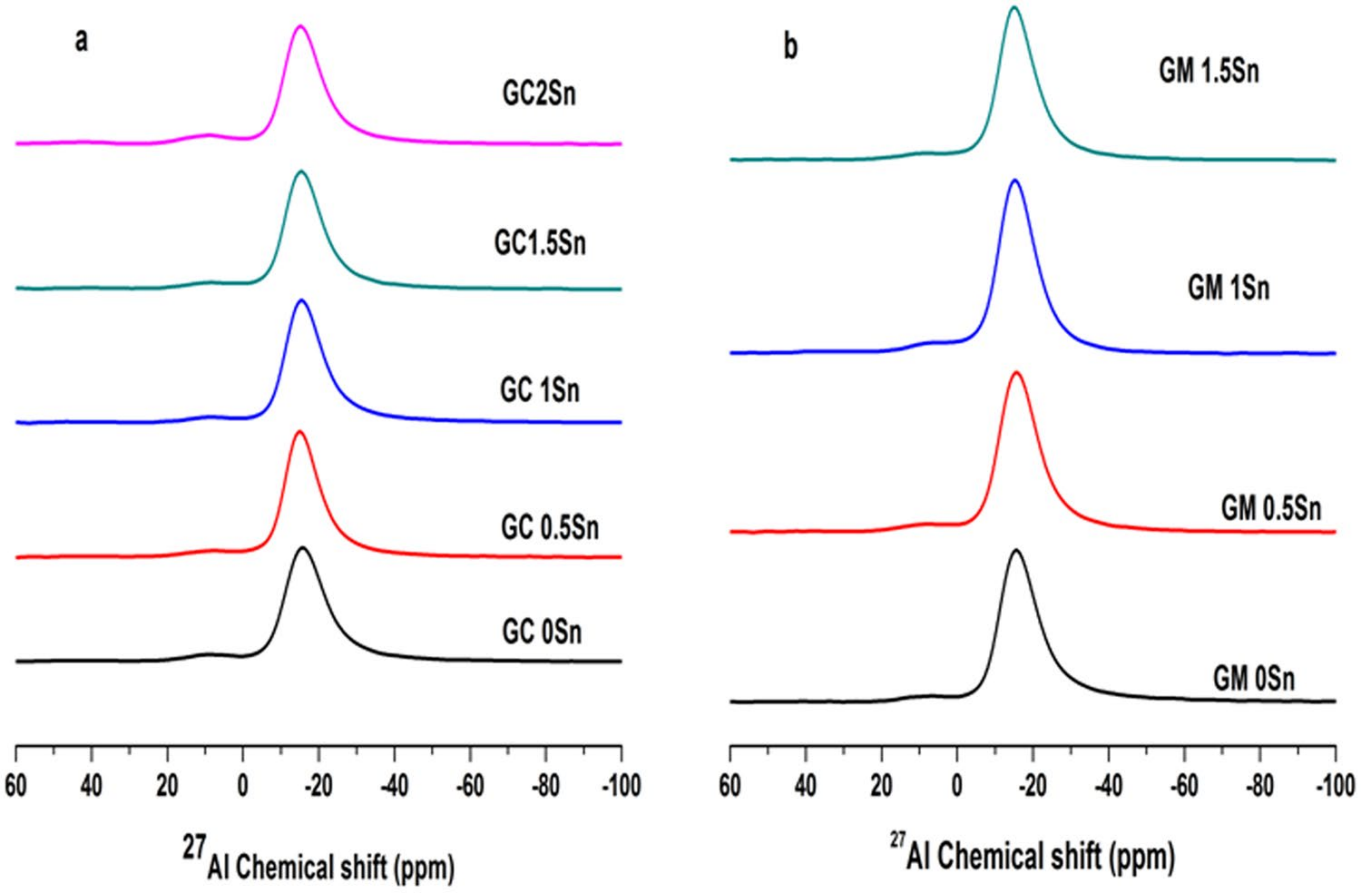
^27^Al MAS NMR spectra for the glasses having different concentration of Sn.; (**a**) conventional heating; (**b**) MW heating.

The ^27^A1 MAS NMR spectra of the samples prepared in both heating are displayed in Fig. 14. The spectra of the glass samples in Fig. 14 show two peaks located at ~14 and ~−14 ppm. A very weak peak can be identified at ~53 ppm. The chemical shift and the relative content of all these sites are listed in the Table 2.

**Table 2:** Relative content of Al sites and their chemical shift obtained from ^27^Al MAS NMR spectra after deconvolution.

Glass	Chemical shift (ppm)	% of occupancy	Glass	Chemical Shift(ppm)	% of occupancy
GC0Sn	53.32	0.97	GM0Sn	45.79	0.82
	14.21	7.03		14.15	4.24
	−14.29	91.99		−14.60	94.94
GC0.5Sn	55.23	1.16	GM0.5Sn	53.86	1.16
	14.21	9.41		14.30	7.01
	−14.40	89.43		−14.63	91.83
GC1Sn	52.56	0.89	GM1Sn	53.98	0.94
	15.86	5.59		14.51	4.14
	−14.60	93.52		−14.56	94.92
GC1.5Sn	53.59	2.08	GM1.5Sn	56.67	0.28
	16.12	6.39		15.22	4.86
	−14.51	91.53		−14.44	94.86
GC2Sn	55.46	0.83			
	14.59	4.62			
	−14.94	94.54			

## Discussion

Although, the glasses without reducing agent are brown in colour for both the heating, darker colour appears in conventional glass as seen in Fig. 1. Colour of sample GM 0.5Sn (glass containing 0.5 wt.% of Sn metal) has been reduced significantly in MW heating. Further, addition of 1wt.% Sn in batch makes the glass visibly colourless in MW heating. However, all the four conventional glasses are observed to be brown in colour. This indicates that effect of reducing agent (Sn) is enhanced in MW heating than in conventional heating in making colourless phosphate glass. Observation of a broad hump and absence of any sharp peak in XRD profile presented in Fig. 2, confirm amorphous nature of the samples. This further reveals that in MW heating, no unmelted crystalline phase (raw material) is left within the glass.

In Vis-NIR spectra shown in Fig. 3a, intensity of broad absorbance band centred at ~1050 nm increases with increasing concentration of Sn metal in the glass depicting more retention of of Fe^2+^ in microwave prepared glasses. In contrast, visible improvement of the peak intensity is recorded in conventional glass containing Sn metal >1 wt.% of Sn. Addition of 0.5 wt.% Sn in conventional glass does not improve this intensity causing no visible colour change in the glass (GC 0.5Sn) as appeared in Fig. 1. Sn metal concentration could be optimised ~1 wt.% in MW heating in order to make colourless glass with corresponding Fe[II] absorbance of ***2.31 cm***^***−1***^ as seen in Fig. 4. In conventional heating, addition of 1.5 wt.% Sn metal improves the Fe[II] absorbance to 2.12 cm^−1^. Although visible colour diminishes with 1.5 wt.% of Sn, further addition of Sn metal is necessary to make the glass colourless in conventional heating. Moreover, it can be seen that the nature of increase of Fe[II] absorbance intensity in both heating is found different as shown in Fig. 4. In MW, concave downward curve is noticed, whereas it is concave upward in conventional processing.

In colorimetric analysis, intensity of absorbance at 562 nm in Fig. 5a ascribed as Fe^2+^-Ferrozine colour complex^24,25^, increases with increasing concentration of Sn in glass corroborating enhance formation of Fe[II] in glass with addition of reducing agent. This is in similar agreement with optical absorbance study. Although absorbance intensity at 562 nm increases with increasing concentration of Sn in conventional heating, increase of this intensity for GC 0.5Sn is recorded much less than that of GM 0.5Sn. In microwave heating, it has been observed that absorbance intensity at 562 nm is enhanced further with addition of 1 and 1.5% Sn as depicted in Fig. 5a. In conventional glasses, increase of absorbance intensity of Fe^2+^-Ferrozine complex in the solution is observed noticeable for 1.5 wt.% addition of Sn (i.e. GC 1.5Sn sample) as seen in Fig. 5b.

Fe-redox ratio is evaluated 0.49 for GM 1 Sn sample and the glass containing 1 wt.% of Sn is obtained colourless glass. Thus, this can be predicted that ***Fe-redox ratio 0.49*** (corresponding **Fe[II] absorbance** ***2.31 cm***^***−1***^) *or more* is required to make the glass colourless in the studied composition and this can be achieved by addition of ≥1 wt.% Sn metal in microwave heating. Now, Fe-redox ratio can be determined by curve fitting with following second order polynomial equation (1) for MW prepared glasses [Sn concentration within 0–1.5 wt.%] as shown in Fig. 6:

1 $${\bf{y}}={{\bf{y}}}_{{\bf{m}}}+{{\rm{A}}}_{1}{\bf{x}}+{{\rm{A}}}_{2}{{\bf{x}}}^{2}$$

The parameters, such as intercept (y_m_), A_1_ and A_2_ are listed with respective standard error in Table 3. Fitting parameter R^2^ is found 0.996 which is acceptable. Fe-redox ratio in MW heating follow the equation [1] and the nature of curve obtained is found to be concave downward with negative A_2_.

**Table 3:** Parameter for equation (1) with standard error and R-Square for microwave heating.

Parameter	Value	Standard Error
y_m_	0.27695	0.00602
A_1_	0.46229	0.02865
A_2_	− 0.18309	0.02042
R²	0.996	

 Fig. 7 exhibits no significant improvement of iron redox ratio with addition of 0.5 wt.% Sn metal in glass. However, further addition of Sn (1 and 1.5 wt.%) improves Fe^+2^/∑Fe in glass while the glass containing 2 wt.% of Sn metal is obtained colourless as depicted in the inset of Fig. 7.

The curve fitting was obtained adopting following curve fitting equation to determine Fe-redox ratio with varying Sn metal [within 0–2 wt.%] in conventional glasses.

2 $${\bf{y}}={{\bf{y}}}_{{\bf{c}}}+{{\rm{B}}}_{1}{\bf{x}}+{{\rm{B}}}_{2}{{\bf{x}}}^{2}$$

The parameters like intercept (y_c_), B_1_ and B_2_ are listed with respective standard error in Table 4. The nature of the fitted curve is concave upward with negative B_1_. The fitting parameter R^2^ is 0.99. Sn concentration may be evaluated from the fitted curve in Fig. 7. From the fitted curve, Sn is determined ~1.90 wt.% to yield Fe-redox ratio 0.49 and thus colour due to iron in conventional glass can be eliminated.

**Table 4:** Parameter for equation (2) with standard error and R-Square for conventional heating.

Parameter	Value	Standard Error
y_c_	0.23787	0.00926
B1	−0.01065	0.02685
B2	0.07776	0.01499
R²	0.99029	

In MW processing, Fe-redox ratio in glass follows different correlation than in conventional processing. In studied glasses prepared in MW, Fe redox ratio is found to be higher than that of the corresponding conventional glass having identical Sn.

Fe[II] absorbance with time in glass without containing Sn (in Fig. 8) was fitted with following linear curve fitting correlation:

3 $$y=a+bx$$

The fitting parameter R^2^ is noted 0.99. The slopes of the equation determine the rate of oxidation reaction [Fe^2+^  → Fe^3+^] and this is obtained −0.0043 in microwave and −0.0101 in conventional heating. *Thus, it is apparent* *that formation of Fe[III] is slower in microwave heating than conventional ensuring higher retention of Fe[II] in glass*.

Figure 9 depicts weight gain in both the glasses GMF5 and GCF5 depicting conversion of FeO to Fe_2_O_3_ by using oxygen from the air. Weight gain in TGA curve is due to the conversion of FeO to Fe_2_O_3_ following equation (4).

4 $$FeO+\frac{1}{2}{O}_{2}\to F{e}_{2}{O}_{3}$$

Weight gain is recorded 0.10% in microwave prepared glass and 0.01% in conventional glass. Less weight gain in GCF5 signifies less conversion of FeO to Fe_2_O_3_ compared to the glasses prepared in MW heating (GMF5). This result is found similar to the findings of earlier section. Thus, TGA analysis also confirms more formation of Fe[II] in MW prepared glasses. Conversion of SnO to SnO_2_ may also influence the weight gain during TGA analysis.

Narrow scan XPS spectra, as shown in Fig. 10a, for Sn 3d exhibit two peaks at ~487 eV ascribe to Sn 3d5/2 and ~497 eV for Sn 3d3/2. The peak of Sn 3d5/2 is usually used to determine the oxidation state of Sn in the material. The deconvoluted spectrum of Sn 3d5/2 is shown in Fig. 10b. It can be clearly seen from the Fig. 10b that Sn 3d5/2 peak for conventional glass (GCF5) is shifted towards higher binding energy indicating higher presence of SnO_2_ than in microwave prepared glass. Similarly, narrow scan Fe 2p spectra for GCF5and GMF5are exhibited in Fig. 10C. Two clear peaks at 710 and 723 eV are visible along with a satellite peak for Fe_2_O_3_ at ~715 eV. The XPS band at 710 is assigned to Fe 2p_3/2_ and band at 723 is ascribed to Fe 2p_1/2._ The position of Fe 2p_3/2_ indicates presence of Fe in different oxidation state (such as II or III). However, Fe is in equilibrium with both oxidation states (i.e. FeO and Fe_2_O_3_) in glass. Deconvoluted Fe2p_3/2_ peak at ~710 eV is plotted in Fig. 10d depicting shift of peak towards lower binding energy side for microwave melted glass. In microwave melted glass, the Fe 2p_3/2_ peak center is found to at 710.06 eV whereas it at 710.65 eV for conventional glass. Although it is difficult to distinguish the peak for FeO and Fe_2_O_3_ from this XPS spectra due to overlapping nature of band, it is possible to predict the qualitative presence of the band. Position of Fe 2p_3/2_ band shifting towards lower binding energy side indicates higher concentration of FeO in microwave prepared glass. Thus, presence of Fe 2p_3/2_ peak in lower binding energy in GMF5 (compare to GCF5) confirms higher concentration of FeO in microwave prepared glass.

Temperature, time and power profile suggests that the alternate glass melting using MW heating addresses significant energy efficiency compared to conventional glass melting. Substantial less consumption of energy and shorter processing time have been demonstrated in laboratory scale glass melting. Total power consumption in conventional heating is almost 4 times higher than that of MW heating. Moreover, shorter processing time is also potential to reduce the cost of glass melted in MW heating. This significant reduction in energy consumption would indirectly lead to less generation of green house gases and contribute to a cleaner environment. In conventional heating, furnace cavity is heated along with material signifying more requirement of electrical power. This increases the temperature of atmosphere around the furnace causing uncomfortable work environment and thus, creates material handing difficulties. Direct heating of material in MW creates comfortable work atmosphere, thereby making this technology a green processing technology. In the perspective of inadequate resources to meet future power demand and the present critical review of other potential resource like nuclear energy, all out effort is the need of time to find out product, process, technology which will consume less energy. In this context, MW glass melting process could be a potential energy efficient technology saving significant amount of electrical energy and time, needed for present glass making. In addition to the energy efficiency, MW heating has the potential to alter glass property. Fe-redox ratio can be enhanced in MW heating and heat (IR) absorbing glass can be obtained in air unlike in conventional heating which requires stringent atmosphere control in addition to incorporation of reducing agent in the melt. Further, MW heating can be used to nullify the colour of Fe impurity in phosphate glass.

All the glasses are dominated by the ultra (Q^3^) and meta phosphate (Q^2^) units as seen in the ^31^P MAS NMR spectra (Fig. 13). It is seen that on increasing the content of Sn, the relative contribution of Q^3^ slightly increases at the expense of a gradual decrease in Q^2^ sites. This transition from cross linked ultra phosphate to linear polymer metaphosphate network, clearly indicates the availability of non bridging oxygen sites for the incorporation of Sn^26,27^. This trend is predominant in the case of glasses prepared by conventional method than the MW preparation. Glasses having similar Fe redox ratio have comparable site occupancy for Q^2^ and Q^3^. As the percentage of Sn increases, the chemical shift of the isolated PO_4_^3−^ tetrahedral unit are shifting to lower frequency and their contribution also decrease in glasses prepared by conventional method. But no significant changes are observed for the Q^0^ sites of the GM series except GM0.5. It is noted that the total area (%) of Q^0^ and Q^1^ follows entirely different type of correlation in both the type of glasses. All these findings further corroborate the role of Sn and the mode of heating employed in the glass preparation.

The peaks seen in ^27^A1 MAS NMR spectra (Fig. 14) at ~53, ~14 and ~−14 ppm are assigned to four coordinated, five-coordinated and six-coordinated Al, respectively^28^. The relative content of Al^6+^ is less in glasses prepared by conventional heating in comparison with MW heating having same % of Sn. The percentage of the four coordinated Al is very less in all the glasses. No other significant changes were observed.

## Material and Method

### Material

The studied glass compositions (in wt.%) are mentioned in Table 5. Aluminium-meta-phosphate was chosen as source of raw material for Al_2_O_3_ and P_2_O_5_, Na_2_CO_3_ for Na_2_O and P_2_O_5_ was added to maintain the composition stoichiometry. Sn metal was varied between 0–1.5 wt.% in batch. 2 wt% of Sn metal was added only to estimate iron-redox ratio in conventional glass. The obtained glasses prepared in MW and conventional are marked and mentioned in the Table 5.

**Table 5:** Batch composition, Sn concentration, glass identification in microwave and conventional heating.

Batch Composition	Concentration of Sn	Glass melted in microwave heating (GM)	Glass melted in Conventional heating (GC)
Al_2_O_3_-9, Na_2_O-16, P_2_O_5_-74, Fe-1 (metal)	0	GM 0Sn	GC 0Sn
	0.5	GM 0.5 Sn	GC 0.5 Sn
	1	GM 1Sn	GC 1Sn
	1.5	GM 1.5Sn	GC 1.5 Sn
95*(Al_2_O_3_-8, Na_2_O-15, P_2_O_5_-72) Fe–5	2	GMF5	GCF5

### MW heating

All the raw materials were thoroughly mixed and pelletized with hydraulic press operated within 0–10 ton pressure. This pelletized batch was placed in an alumina crucible. The crucible containing batch was properly insulated with MW transparent thermal insulation box, which possess a hole at the top for monitoring temperature through infra-red (IR) pyrometer and placed within cavity properly aligned with pyrometer. The working range of pyrometer is 260–1800 °C with an accuracy of ±0.3% of measured value +1 °C. The glass was melted at 1150 °C for 30 min and manual stirring was adopted to have homogeneous glass. Temperature, MW output power (MW power) and time were recorded using DAQ software installed in a separate computer. Molten glass was poured into a preheated steel mould and subsequently transferred to an annealing furnace maintained at 320 °C followed by controlled cooling until room temperature.

### Conventional heating

Identical batches were melted in a conventional resistance heating furnace (of cavity dimension diameter 200 mm and 250 mm height). The melting was carried out at 1150 °C for 30 min adopting one time manual stirring. The glasses were annealed as discussed in earlier section. Temperature and electrical power were monitored with time during the melting process. Instantaneous power was recorded operating the furnace in manual mode.

### Characterization

X-ray diffraction (XRD) analysis was performed by 94 X’Pert, PANalytical using Ni-filtered CuKα radiation with wavelength of 1.5406 Å to determine the amorphous phase of the samples. The scanning range was fixed from 5–90° with a step size of 0.05° min^−1^.

Glass sample of dimensions 10 × 15 (in mm) and 0.5 mm thick was prepared from all the glasses prepared by both the heating routes. The optical absorption spectra were recorded on a UV–Vis-NIR spectrophotometer (Model: Perkin Elmer Lambda 950, USA) in the wavelength range 250–1500 nm.

A spectrophotometric method was employed to estimate Fe^2+^ developing Fe^2+^-Ferrozine colour complex in the acid solution (H_2_SO_4_ and HF). Meta-NH_4_VO_3_ was added to prevent aerial oxidation of Fe^2+^. In order to identify total iron in the solution, 5% ascorbic acid (Merck, Darmstadt, Germany, 99% pure) solution was used to reduce all the iron to Fe^2+^ state, followed by the addition of ferrozine-buffer solution to develop the Fe^2+^-ferrozine colour complex. Now, intensity of optical absorbance of Fe^2+^-Ferrozine colour complex is the measure of total iron in the solution. Fe- redox ratio was estimated from the absorbance intensity of Fe^2+^-ferrozine colour complex in the solution without and with the addition of ascorbic acid. The optical absorbance of the solution containing Fe^2+^-ferrozine colour complex having broad absorption band at 562 nm was recorded by UV–Vis spectrophotometer (Model Perkin Elmer, Lambda 45, USA) within the wavelength range 450 to 550 nm. The intensity of optical absorbance of Fe^2+^-Ferrozine colour complex (at 562 nm) is the measure of Fe^2+^ in the solution. Three successive measurements were carried out and accuracy was found to be within 2–3%. Details of this experimental method, accuracy and precision of measurement have been described elsewhere^29^.

Thermo gravimetric analysis (TGA) was performed using ~80 mg of glass powder in a standard Pt-Rh crucible by simultaneous thermal analyzer *NETZSCH STA* 449 C. TGA analysis was performed for glass obtained from melting batch containing 5 wt.% Fe metal in both heating (of GCF5 and GMF5). The experiment was performed at a heating rate of 10 K/min until 800 °C and kept for 30 min. The glass was held at 150 °C for 30 min to evaporate the loosely bonded OH, absorbed gas and moisture. The experiment was performed under flowing air at 40 LPM.

X-ray photoelectron spectroscopy (XPS) measurements were carried out using glass samples of GCF5 and GMF5 using PHI 5000 XPS-analyzer, Versaprobe-II, USA. The Al-Kα source (1486.6 eV) and a hemispherical analyzer with 16-channel detector was located at 54.7°position with respect to the sample surface (analyzer axis) and run at X-ray setting 200 μ 25 W/15 kV with the pressure 5 × 10^−8^ Pa inside the vacuum chamber and recorded via smart soft-Versaprobe 2.4.0.9 software. These spectra were fitted to Gaussian–Lorentzian distribution using origin 8.5 software to determine the core level binding energy of Fe2p, Sn3d.

The ^31^P and ^27^Al single pulse NMR spectra were collected on a Bruker AVANCE 700 MHz spectrometer equipped with a 1.3 mm probe at a sample rotation frequency of 60 kHz. The Larmor frequency for ^31^P and ^27^Al were 283.418 Hz and 182.432 Hz respectively. In the ^31^P Magic angle spinning (MAS) NMR, a 90° pulse of duration of 1μs and a recycle delay of 10 s was employed. The ^27^Al MAS NMR spectra were acquired using small flip-angle pulses of 0.2μs length with a recycle delay of 2 s. A total of 256 scans for ^31^P and 12000 scans for ^27^Al were acquired. 85% of aqueous phosphoric acid and 1 M aluminium nitrate solution were used as a chemical shift reference for ^31^P and ^27^Al respectively. The ^31^P and ^27^Al MAS NMR signal deconvolutions into components were carried out using the DMFIT program^30^.

### Data availability statement format guidelines

Additional data generated during the study is available with the corresponding author.

## Conclusion

Effect of MW heating has been studied to produce colourless phosphate glass with high heat absorbing properties. **Fe-redox ratio** of **0.49** is found to be the threshold for preparation of colourless glass. Sn concentration of 1 wt.% is optimized to obtain colourless phosphate glass in MW heating. The addition of Sn metal of ~1.9 wt.% can yield desired Fe-redox ratio to make the glass colourless in conventional heating. The correlation equations developed for MW and conventional heating are found to be different. In MW, the nature of curve for Fe-redox ratio with varying Sn concentration is concave downward unlike concave upward in conventional. Slow formation of Fe[III] in microwave heating enhances higher stabilisation of Fe[II] in glass than that in conventional heating. All the glasses are dominated by the ultra (Q^3^) and meta phosphate (Q^2^) units as seen in the ^31^P MAS NMR spectra. Transition from cross linked ultra phosphate to linear polymer metaphosphate network takes place for incorporation of Sn. This trend is predominant in conventional glasses than the microwave preparation. ^27^A1 MAS NMR spectra suggest that relative content of Al^6+^ is higher in glass obtained from MW heating. Significant reduction in energy consumption would indirectly leads to less generation of greenhouse gases enabling microwave glass melting a green and clean processing. Thus, energy efficient MW heating plays a significant role in improving properties in glass. Particularly, microwave processing produces enhanced heat (IR) absorbing properties which can be of use in preparing IR absorbing glass in air atmosphere, which is not feasible in conventional heating.
